# Rare Human Diseases: Model Organisms in Deciphering the Molecular Basis of Primary Ciliary Dyskinesia

**DOI:** 10.3390/cells8121614

**Published:** 2019-12-11

**Authors:** Martyna Poprzeczko, Marta Bicka, Hanan Farahat, Rafal Bazan, Anna Osinka, Hanna Fabczak, Ewa Joachimiak, Dorota Wloga

**Affiliations:** 1 Laboratory of Cytoskeleton and Cilia Biology, Nencki Institute of Experimental Biology of Polish Academy of Sciences, 3 Pasteur Street, 02-093 Warsaw, Poland; m.poprzeczko@nencki.edu.pl (M.P.); m.bicka@nencki.edu.pl (M.B.); h.farahat@nencki.edu.pl (H.F.); r.bazan@nencki.edu.pl (R.B.); a.osinka@nencki.edu.pl (A.O.); h.fabczak@nencki.edu.pl (H.F.); e.joachimiak@nencki.edu.pl (E.J.); 2 Faculty of Chemistry, University of Warsaw, 1 Pasteura Street, 02-093 Warsaw, Poland

**Keywords:** ciliopathies, motile cilia, *Chlamydomonas*, zebrafish, *Xenopus*, mouse

## Abstract

Primary ciliary dyskinesia (PCD) is a recessive heterogeneous disorder of motile cilia, affecting one per 15,000–30,000 individuals; however, the frequency of this disorder is likely underestimated. Even though more than 40 genes are currently associated with PCD, in the case of approximately 30% of patients, the genetic cause of the manifested PCD symptoms remains unknown. Because motile cilia are highly evolutionarily conserved organelles at both the proteomic and ultrastructural levels, analyses in the unicellular and multicellular model organisms can help not only to identify new proteins essential for cilia motility (and thus identify new putative PCD-causative genes), but also to elucidate the function of the proteins encoded by known PCD-causative genes. Consequently, studies involving model organisms can help us to understand the molecular mechanism(s) behind the phenotypic changes observed in the motile cilia of PCD affected patients. Here, we summarize the current state of the art in the genetics and biology of PCD and emphasize the impact of the studies conducted using model organisms on existing knowledge.

## 1. Introduction

The cilium is an ancient eukaryotic organelle, believed to be present in the last eukaryotic common ancestor (LECA) [1]. Nowadays, cilia are ubiquitously assembled by numerous evolutionarily distant unicellular eukaryotes and differentiated cells of multicellular organisms, including humans. Based on the functional and ultrastructural differences, cilia can be divided into two groups: immotile and motile organelles. 

Generally, the immotile cilia are assembled as a single organelle (called the primary cilium) by non-dividing cells (G1 or G0 phase). However, some types of cells have more than one immotile cilium (e.g., olfactory sensory neurons form up to 30 immotile cilia [2]). Because of the presence of specific receptors in the ciliary membrane, immotile cilia function as sensors: They “receive” the extracellular signals from the surrounding environment and, after the activation of the receptor, signaling molecules are transmitted to the cell body [3]. Consequently, the primary cilia play a role in the determination of the cell fate, tissue and organ architecture, and body development [4,5]. In mammals, including humans, the primary cilia are assembled by nearly all types of cells [6,7].

In contrast, motile cilia are usually assembled as multiple structures. They can also perform sensory functions [8,9], but their primary role is to propel free-living organisms and sperm cells and to shift extracellular fluids and particles along the surface of the ciliated epithelial cells. 

In humans, motile cilia are restricted to sperm cells (a single long cilium called the flagellum or sperm tail) and epithelial cells lining the nasal cavity, paranasal sinuses, middle ear, respiratory tracts, brain ventricles, and the Fallopian tube. The coordinated asymmetric movement of motile cilia enables the expulsion of mucus, inhaled particles, and bacteria out of the respiratory system and the circulation of the cerebrospinal fluid through the brain ventricular system, and contributes to the transport of the oocyte and early embryo in the Fallopian tube [10]. A specific type of motile cilia—so-called nodal cilia—are formed by the node during embryonic development. The nodal cilia are assembled as a single organelle. They lack some motile cilia-specific structures (a central apparatus and radial spokes (see below)). As a consequence, nodal cilia perform symmetric rotatory movement that generates the left-directed nodal flow, initiating the formation of the left–right asymmetry in the arrangement of the main internal organs [11].

Considering that cilia are involved in a broad spectrum of biological processes, it is not surprising that, in humans, the lack or assembly of shorter, fewer, or improperly functioning cilia manifest as multi-symptom disorders collectively called ciliopathies. The vast majority of known ciliopathies are caused by the dysfunction of the primary cilia, while defects in motile cilia result in a syndrome called primary ciliary dyskinesia (PCD, [MIM: 244400]). The knowledge of the molecular basis of human ciliopathies has significantly advanced within the last several years. Such progress would not be possible without the extensive analyses of the mechanisms controlling cilia assembly and function conducted using diverse model organisms. Here, we emphasize and discuss the importance of studies conducted using model organisms in uncovering the molecular basis of the primary ciliary dyskinesia. 

## 2. Ciliary Ultrastructure

**Figure 1:**
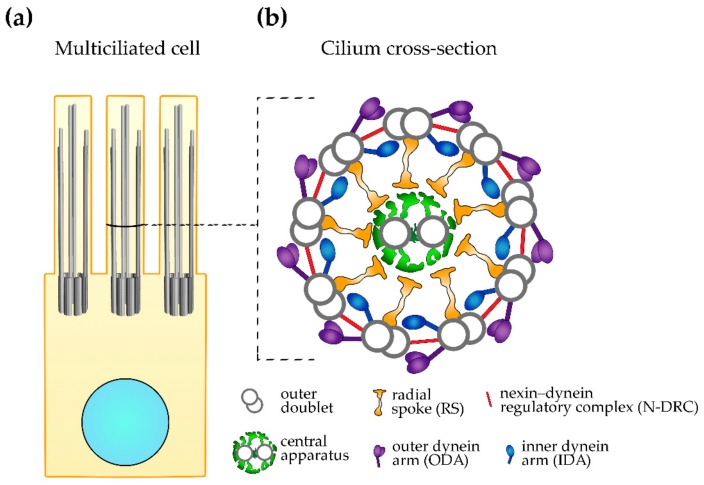
Motile cilium ultrastructure. (**a**) A schematic representation of the multiciliated cell with marked nucleus (light blue), basal bodies, and cilia. (**b**) A schematic representation of the cilium cross-section, showing large ciliary complexes (the level of the cross-section marked on (**a**).

The ultrastructure of cilia has been strikingly well conserved throughout evolution. A core (an axoneme) of both primary and motile cilia is composed of nine microtubular doublets positioned at the cilium periphery; these microtubules are continuous with two out of three microtubules of nine microtubular triplets of the basal body—a structure that anchors cilium to the cell body and docks to the cell membrane through transition fibers. The core of motile cilia (and immotile cilia assembled by the specific cell types [2]) additionally contains two centrally positioned single microtubules: C1 and C2 (9 + 2 configuration, Figure 1) [12,13]. Motile cilia also have numerous multi-protein complexes that are attached to both peripheral and central microtubules [12,13]. The central microtubules together with their complexes, so-called projections (C1a–C1f and C2a–C2e), and a complex connecting both microtubules (a bridge) form a central apparatus [14]. It is believed that part of the signals regulating cilia beating originate in this central structure [13].

The outer doublet microtubules are docking sites for four types of large complexes: outer and inner dynein arms (ODAs and IDAs, respectively), nexin–dynein regulatory complexes (N-DRC) and radial spokes (RSs), and numerous small complexes of mostly unknown protein composition and function [12,15,16]. These large complexes, as well as small ciliary complexes, are arranged along the peripheral microtubules, forming a characteristic pattern composed of 96 nm units called the axonemal repeats; each repeat has four identical ODAs (two- or three-headed, depending on the organism), seven IDAs (one two-headed and six single-headed, all containing different dyneins), a single N-DRC, three RSs that differ slightly in their structure and likely functions, and a single copy of the small complexes (e.g., tether/tetherhead, MIA complex) [15,16,17,18,19]. 

The radial spokes extend in the direction of the central apparatus. It was proposed that the transient contacts between the radial spokes and projections of the central apparatus facilitate the transmission of the signals that originate at the central apparatus to the outer doubles and dynein arms, thus regulating cilia beating [20]. 

The outer and inner dynein arms are formed by numerous protein subunits, including dynein arm type-specific minus end-directed motors, dyneins. The spatio-temporally synchronized movement of the activated dyneins along the adjacent microtubule results in a shift of the neighboring doublets [21]. The range of this shift is limited by the N-DRC, which extends between two neighboring doublets. Consequently, the shift of the outer doublets is translated into a cilium bend, and subsequent bends into cilium beating.

The large outer doublet complexes can be viewed using a classical transmission electron microscope (TEM), but small complexes or small alterations in the architecture of large complexes are below the TEM detection level.

## 3. Primary Ciliary Dyskinesia (PCD)

Based on the proteomic and genomic data, it is assumed that the assembly and proper functioning of motile cilia require up to several hundred proteins [22,23,24]. Mutations in genes encoding ciliary proteins can result in a lack of cilia, the assembly of shorter or fewer cilia, or alterations in cilia function. Until now, mutations in more than 40 genes have been shown to cause PCD. However, it is estimated that the genes identified to date account for only 60–70% of all PCD cases [25]. 

Primary ciliary dyskinesia affects approximately one in 15,000–30,000 individuals, but this condition is likely underdiagnosed [26]. It manifests mainly by cough, sputum production, rhinitis, sinusitis, otitis media, hearing impairment, and recurrent infections of the upper and lower respiratory tracts caused by impaired mucociliary clearance. As the disease progresses, patients can suffer from irreversible lung damage and, in extreme cases, can even require lung transplantation [27]. A significant number of male patients are infertile due to the reduced motility or immotility of the sperm cells. In females with PCD, the transport of the ovum in the Fallopian tube can be slowed down, and this can lead to an ectopic pregnancy and infertility [28,29,30]. Some PCD patients suffer also from congenital heart disease and, rarely, scoliosis, retinitis pigmentosa, and hydrocephalus [30,31]. In about half of PCD patients, the arrangement of the main internal organs in the chest and abdomen is reversed (situs inversus) or, rarely, abnormal (situs ambiguus) due to the dysfunction of the nodal cilia [32]. However, these abnormalities are never observed in patients with mutations in genes encoding subunits of the radial spokes or central apparatus (as human nodal cilia lack these structures). 

The severity of the PCD symptoms depends upon the extent of the alterations of cilia structure and function, and those depend upon the mutated gene. For example, cilia in individuals with PCD caused by the mutation in *CCDC39* or *CCDC40* (that results in various ciliary defects, see Section 5.2) or *DNAH5* (encoding ODA γ dynein heavy chain, Section 5.3.1) were immotile, while the mutation in *DNAH9* encoding β dynein heavy chain of ODAs present in the distal part of cilia caused only a slightly reduced bending of the distal part of cilia (see Section 5.3.1). Moreover, neonatal distress, otitis media, or bronchiectasis were not reported in patients with *DNAH9* mutation (although the group of PCD patients in this study was small) [33], while such symptoms were very frequent in individuals with mutations in *CCDC39*, *CCDC40,* or *DNAH5,* see Supplementary Materials [34,35]. 

Theoretically, mutations (pathological variants) in genes encoding proteins building the core region of the large complex (e.g., DRC1 or DRC2, see Section 5.4) or docking the entire complex to the axoneme should cause more severe damage to cilia motility than mutations in proteins positioned at the complex periphery (unless this peripheral protein/region of the complex is involved in the protein–protein interactions crucial for the biological process). Indeed, mutations in genes encoding the ODA docking complex have a stronger effect on cilia than mutations in *DNAH9*. 

As was shown in studies using model organisms, some mutations can cause small changes in cilia beating [36]. Thus, it is possible that individuals harboring similar mutations have no symptoms or manifest only mild symptoms and are never diagnosed for PCD. 

Because PCD is a genetic disorder caused by mutations in a number of different genes and the severity of symptoms can vary in affected individuals, the diagnosis of this ciliopathy might be a challenge. The measurement of the level of production of nasal nitric oxide (which is usually reduced in individuals affected by PCD) is a basic screening test. The measurements of the cilia beating (frequency, amplitude, and cilia synchrony) using high-speed video-microscopy (HSVM), detection of marker proteins (e.g., DNAH5) using immunofluorescence microscopy and specific antibodies, and identification of the ultrastructural defects using transmission electron microscopy (TEM) in respiratory epithelial cells obtained from patients by nasal brush biopsy are other diagnostic tests that are conducted in the case of individuals with clinical symptoms to confirm cilia dysfunction. The genetic tests can give a clear-cut result, pointing also to the causative gene [37,38,39]. 

The number of targets in the genetic tests is limited by the number of known PCD-related genes. The identification of new PCD causative genes and, subsequently, the enrichment of the library of genes that should be tested in individuals with symptoms pointing to PCD can be achieved directly by next-generation sequencing of DNA samples obtained from affected individuals, or indirectly by deciphering the role of novel ciliary proteins in the generation and/or regulation of cilia beating using model organisms. 

## 4. Advantages of the Model Organisms

The amount of the biological material obtained during a patient’s biopsy is limited, and thus is often insufficient for detailed biochemical and molecular analyses. Because cilia are highly conserved in eukaryotic cells not only at the ultrastructural level, but also at the molecular level, such analyses can be performed using model organisms. The vast majority of the ciliary proteins identified to date are ubiquitously expressed in ciliated cells from protists to humans. Thus, not surprisingly, the current comprehensive knowledge regarding ciliary proteins, their precise localization and function in cilia were obtained using diverse model organisms. Moreover, research conducted using model organisms can help us to understand the molecular basis of the ciliary defects triggered by PCD-causative mutations. Several unicellular and multicellular organisms have emerged as models to study ciliopathies, for review [40,41,42,43,44].

### 4.1. Unicellular Models—the Power of Being Small

Besides the biflagellated green algae *Chlamydomonas reinhardtii*, which is unquestionably a leader unicellular model in the field of cilia biology, several other organisms, including ciliates *Tetrahymena thermophila, Paramecium* sp., and parasitic kinetoplastid *Trypanosoma* sp., are used to study cilia biology. Compared to the vertebrate models, the culturing of unicellular organisms is relatively easy, inexpensive, and fast due to the short generation time. 

The unicellular models differ in the number and length of the assembled cilia or flagella. *Trypanosoma* has a single, approximately 20-μm-long flagellum. The new flagellum is assembled early during cell division, and thus new and old flagella co-exist in a dividing cell (which is very useful in some types of experiments). *Chlamydomonas* flagella are about 12 μm long. *Tetrahymena* assembles up to 500 approximately 6-μm-long cilia and *Paramecium* assembles up to 1000 slightly longer cilia. The established deciliation methods and availability of a sufficient amount of biological material in the case of unicellular models are advantageous in large-scale biochemical studies, including proteomic analyses. Moreover, the fully sequenced genomes and availability of the reverse genetic tools enable gene knock-out or knock-down, gene replacement, directed mutagenesis, endogenous tagging, and overexpression. Unicellular organisms are also excellent models to analyze cilia beating patterns, including the ciliary waveform, amplitude and beating frequency, the distance traveled by cells, and cell trajectories [45,46,47,48,49,50]. 

The availability of a sufficient number of cilia also expedites the analyses of defects in cilia ultrastructure using classical TEM and cryo-electron tomography (cryo-ET), followed by three-dimensional (3D) reconstruction—a method that facilitates the better preservation of the biological material and analyses at a higher resolution [51,52]. The cryo-ET studies of the *Chlamydomonas* and *Tetrahymena* axonemes revealed details of the architecture of known large ciliary complexes, uncovered new previously undetected small ciliary complexes, and helped to establish the localization of the protein subunits within multi-protein ciliary complexes—and thus significantly advanced the current knowledge of cilia structure. 

### 4.2. Vertebrate Models

Not all questions concerning cilia assembly and functioning can be addressed in unicellular organisms. Vertebrate models enable studies on cilia in the context of tissue. The unquestionable advantage offered by the vertebrate models is the option to investigate the role of cilia in the embryonic development and postnatal processes at the levels of tissues, organs, and the whole organism. 

Zebrafish (*Danio rerio*), frog (*Xenopus leavis),* and mouse are well-established models to verify the molecular basis of PCD. Both zebrafish and *Xenopus* females lay a large number of eggs (up to 500 and 2000, respectively). The fast embryonic development that takes place entirely outside the maternal organisms, the large number of siblings supporting comparative analyses, and, in the case of zebrafish, the transparency of the embryo body enabling in vivo cilia analyses make these organisms models of choice to analyze PCD-linked genes. 

In zebrafish, the motile cilia can be observed as soon as 11–13 h post-fertilization in the Kupffer’s vesicle, a structure functionally corresponding to the mammalian embryonic node. Several hours later, cilia can be also observed in other locations, including pronephric ducts and brain ventricles [53,54]. Mutations in genes affecting cilia assembly or function frequently result in the characteristic curly body shape of the developing embryo, laterality defects caused by the dysfunction of cilia in the Kupffer’s vesicle, heart looping, perturbation of otolith formation in the inner ear, the formation of cyst in kidneys, and hydrocephalus [42,53,55,56]. 

In *Xenopus*, cilia can be first observed about 18–20 h post-fertilization in a structure called a gastrocoel roof plate that functionally corresponds to the mammalian node [57]. Approximately 12 h later, cilia are assembled by the multiciliated cells present in the skin of *Xenopus* embryos. Interestingly, due to the presence of both multiciliated cells and mucus-secreting goblet cells, the embryo’s skin, to some extent, resembles the epithelium lining the mammalian respiratory tracts [58]. Equally importantly, in the case of both models, the microscopic methods are well developed, and gene manipulation and genome editing are possible [44,59,60,61,62,63,64,65].

In comparison to zebrafish and *Xenopus*, experiments on mice require more time and are more expensive. However, a well-established method to generate the conditional mutants using a Cre-lox system and the high similarity of mouse and human genomes, as well as the four-chambered heart—as with the human heart—make the mouse model an appreciated model in the study of the molecular basis of ciliopathies, including PCD [66]. 

## 5. Causative Genes and Ultrastructural Changes

**Figure 2:**
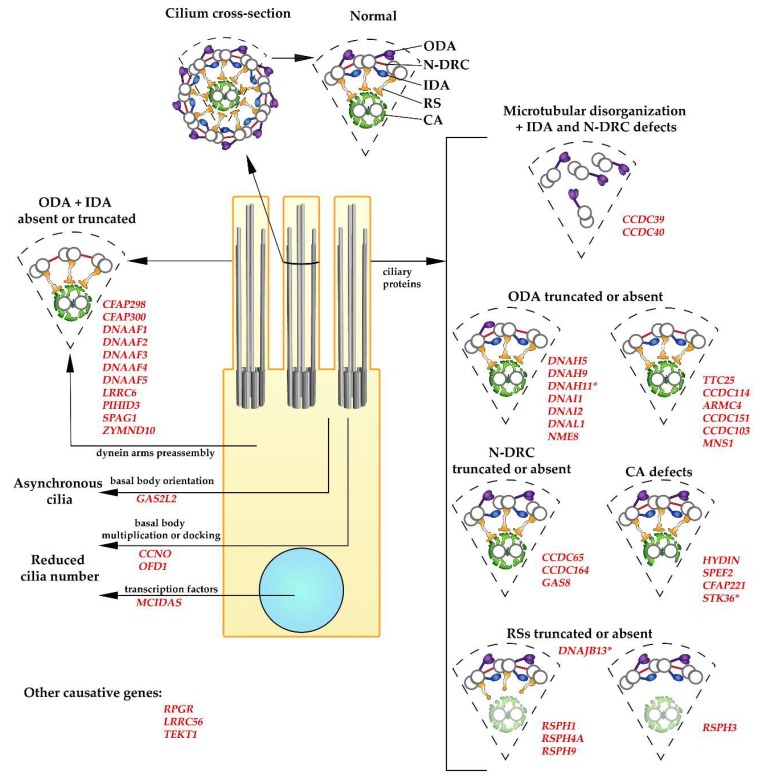
Ultrastructural defects caused by mutations in primary ciliary dyskinesia (PCD)-causative genes. The diagrams show a cross-section of the normal motile cilium with main large complexes and fragments of the axonemes with specific ultrastructural defects observed in PCD (as described on the figure and in the main text). Large complexes: ODA (outer dynein arm, violet), IDA (inner dynein arm, dark blue), RS (radial spoke, orange), N-DRC (nexin–dynein regulatory complex, red), CA (central apparatus, green; the shadow of CA in the diagrams representing structural changes in cilia with mutations in radial spoke proteins illustrates that CA can be missing). Names of the causative genes are in red. * Differences in the ultrastructural changes: *DNAH11*: minor structural defects detected using cryo-ET; *MNS1*: reduces number of the assembled ODAs; *STK36*: abnormalities in CA are rare (~5%); *DNAJB13*: minor structural defects in the radial spoke neck detected using cryo-ET.

PCD is an autosomal (rarely X-linked, e.g., the PIH1D3 gene, see below), recessive, genetically heterogeneous disorder caused by mutations in genes encoding proteins which are indispensable for the assembly and efficient functioning of motile cilia (Figure 2). To date, PCD-causative mutations have been identified in more than 40 genes (Table S1). 

### 5.1. PCD Caused by a Reduced Number of Cilia

Mutations in *MULTICILIN*/*MCIDAS* (Multiciliate Differentiation and DNA Synthesis Associated Cell Cycle Protein) [MIM: 614086] and *CCNO* (Cyclin O) [MIM: 607752] significantly reduce the number of assembled cilia (proposed name: Reduced generation of multiple motile cilia disease, RGMC). Individuals with mutations in these genes have mucociliary clearance disorder, similar to individuals affected by PCD. Detailed analyses of the respiratory epithelial cells obtained during biopsies revealed that, in individuals with a mutation in *MCIDAS*, the number of basal bodies is significantly reduced, and among those that are present in the cell, some basal bodies are not docked to the apical cell surface. In consequence, respiratory epithelial cells are devoid of cilia or rarely assemble only one to two cilia. Moreover, the assembled cilia are immotile and lack key ciliary proteins such CCDC39 and DNAH5 [67]. The analyses of the tracheal epithelial cells from *Mcidas* mutant mice revealed the presence of a single basal body associated with a single cilium, but containing RSPH1, RSPH9, and CCDC40, proteins specific for motile cilia [68]. 

Similar, displaced, and less-numerous basal bodies are observed in the respiratory epithelial cells obtained during biopsies from individuals with mutations in *CCNO*, but in this case, the assembled sparse cilia have normal ultrastructure and beating pattern [69,70]. Morpholino-based depletion of the CCNO in *Xenopus* resulted in reduced numbers of motile cilia [69].

The experiments conducted in model organisms revealed that both proteins play a role in the regulation of centriole amplification during the differentiation of multiciliated cells [71,72]. During airway differentiation, the level of NOTCH determines the fate of the post-mitotic progenitor cells. The inhibition of NOTCH initiates the transcriptional program specific to multiciliated cells, leading to the massive amplification of the centrioles, their migration to the apical cell surface, uniformly oriented docking to the cell surface and ciliation. *MCIDAS* is one of the genes represed by NOTCH [71]. MCIDAS, together with transcriptional factors E2F4/5 and DP1, form a complex which activates, among others, an atypical cyclin, CCNO (cyclin O), which is required during centriole amplification, for review [73,74,75,76]. 

### 5.2. CCDC39 and CCDC40

CCDC39 [MIM: 613798] and CCDC40 [MIM: 613799], the coiled coil domain-containing proteins, are the most intriguing ciliary proteins among proteins whose mutations cause PCD with severe ciliary defects. The phenotypic and ultrastructural alterations caused by the loss-of-function mutation(s) in either gene are basically indistinguishable [77]. The high-speed video microscopy analyses of ciliated cells obtained by nasal brushing biopsy from affected individuals revealed that cilia are either immotile or exhibit some residual motility with rigid beating and a reduced beating amplitude [78,79]. The TEM ultrastructural observations detected various ciliary defects, including mispositioned peripheral doublets with only eight doublets at the cilium periphery and one shifted to the cilium center, displaced central microtubules, absent IDAs, defective RSs, and N-DRC. Interestingly, the localization of ODAs is normal [34,78,79]. Phenotypic alterations consistent with motile cilia defects were also observed in zebrafish embryos and mice embryos with mutated CCDC39 or CCDC40 [79,80]. 

The displacement of the ciliary microtubules is likely a secondary effect—a consequence of the reduced number or lack of the nexin–dynein regulatory complexes that span the adjacent peripheral doublets and defects of the radial spokes. However, the puzzling question is how a loss-of-function mutation in either *CCDC39* or *CCDC40* could result in a lack or the defect of three different ciliary large complexes (IDAs, RSs, and N-DRC).

The localization and functional studies of the CCDC39 and CCDC40 orthologs, FAP59 and FAP172, respectively, in *Chlamydomonas reinhardtii* [81] shed light on the molecular basis behind such multi-structural ciliary defects caused by *CCDC39* and *CCDC40* loss-of-function mutations. Flagella assembled by *fap59* and *fap172 Chlamydomonas* mutants are immotile and lack inner dynein arms and some nexin–dynein regulatory complexes, while the radial spokes are present but exhibit an irregular arrangement along the peripheral doublets. Moreover, as previously observed in PCD patients with mutations in *CCDC39* or *CCDC40*, the ODAs are present in *fap59* and *fap172 Chlamydomonas* mutants [81]. Combined genetic and localization studies using cryo-ET revealed that, in *Chlamydomonas*, FAP59 and FAP172 form a thin, approximately 96-nm-long complex that repeats along the peripheral microtubule doublets and likely functions as (1) a “molecular ruler” defining the length of the axonemal unit, and as (2) a determinant of the docking sites for some ciliary complexes. The expression of the FAP59 and FAP172 proteins, each with duplicated corresponding fragments (N-terminal, middle, or C-terminal fragment duplicated in both proteins) in *fap59fap172* double knockout mutants, results in the assembly of flagella with longer axonemal units and additional large complexes. The length of the formed oversized units is proportional to the number of the duplicated amino acids, while the type of the duplicated complexes depends upon the position of the duplicated fragment (N-terminal, middle, or C-terminal) of FAP59 and FAP172 [81]. Based on the above observations, the authors postulated that the position of RSs and docking of N-DRC and IDAs—but not ODAs—is determined by the FAP59/FAP172 complex. 

Based on the data obtained in green algae, it seems that the extent of the defects in the cilia ultrastructure that translates into cilia immotility and the severity of the PCD symptoms in patients with mutated *CCDC39* or *CCDC40* genes is a consequence of the dysfunction of the mutated proteins as a determinant of the docking/positioning of three important ciliary complexes.

### 5.3. Dynein Arms

A significant number of PCD cases is caused by mutations in genes encoding (1) the subunits of the outer dynein arms, (2) proteins forming the ODA docking complex, or (3) cytoplasmic proteins required for the dynein arm preassembly and the transport of the preassembled dynein arms from the cell body to cilia. 

#### 5.3.1. Dynein Arm Subunits

In human motile cilia, the outer dynein arms are two-headed structures, containing two dynein heavy chains (DHC), γ and β, two intermediate chains, and several light chains [82]. In the epithelial cells lining respiratory tracts, the γ DHC is encoded only by *DNAH5* [MIM: 603335], while β DHC is encoded by one of two genes: *DNAH11* [MIM: 603339] or *DNAH9* [MIM: 603330]. ODAs containing DNAH11 β DHC are present in a more proximal part of cilia (ODA type 1), while ODAs containing DNAH9 are docked in the distal part of cilia (ODA type 2) [83,84]. Out of these three DHCs, mutations in *DNAH5* cause the most severe alterations in cilia beating patterns, while mutations in *DNAH9* result in the mildest alterations [84]. Cilia of the respiratory epithelial cells from patients with mutations in *DNAH5* are either immotile or exhibit residual twitching movement [85,86]. In contrast, mutations in *DNAH11* only reduce the cilia beating amplitude and increase beating frequency [87,88,89], while mutations in *DNAH9* do not alter the beating frequency but only slightly reduce the bending of the distal part of cilia [33,84]. The TEM examination of the respiratory epithelial cells revealed that ODAs are missing along the entire cilia length in patients with *DNAH5* mutations [35,85,86], but only in the distal part of cilia, if *DNAH9* is mutated [33,84] (the distal and proximal parts of cilia are defined based on the absence or presence of the cross-section of the microvilli). In the case of mutations in *DNAH11,* the ultrastructural defects in ODAs cannot be detected using classical TEM [87,88,90]. However, a re-examination using TEM tomography revealed small ODA defects in the proximal part of cilia [90,91]. 

Cilia with missing or truncated ODAs are also a characteristic feature of PCD, caused by mutations in the genes encoding the dynein intermediates chain, *DNAI1* [MIM: 604366] [92,93,94] and *DNAI2* [MIM: 605483] [95], and mutations in the dynein light chain genes *DNAL1/LC1* [MIM: 610062] [96] and *NME8/TXNDC3* [MIM: 607421] [97]—a thioredoxin-like protein with similarity to *Chlamydomonas* LC3 and LC5 [98]. 

The protein composition and role of the dynein arms were extensively studied in diverse model organisms. Within a single axonemal unit, there are four identical ODAs and seven IDAs (a single two-headed IDAf/I1 and six single headed IDAs a to e, and g) that differ in their protein composition and likely function [99]. The phenotypic analysis of the *Chlamydomonas* dynein arm mutants revealed that IDAs and ODAs perform different functions. It is proposed that IDAs control the size and waveform of the cilia bend, while ODAs control the cilia beat frequency [100,101,102]. In contrast to vertebrates that have two-headed ODAs, in model organisms such as *Chlamydomonas* and *Tetrahymena*, ODAs are tri-headed. The analyses of the *Chlamydomonas* cells with mutations in genes encoding ODAs subunits revealed that in the vast majority of studied mutants, ODAs are completely lost [98]. Thus, all studied subunits are most likely required for the presence of ODAs in cilia. The mutations in some of the genes encoding ODA subunits were shown to cause PCD-like symptoms also in mice [103,104,105].

#### 5.3.2. Dynein Docking Proteins

The ODAs are distributed regularly, every 24 nm, along the outer doublet microtubules, except for the cilium distal end [15]. The attachment of ODAs to the microtubules involves a set of proteins forming a docking complex. Mutation(s) in genes encoding subunits of the ODA docking complex result in the assembly of cilia devoid of ODAs. The whole-exome or whole-genome sequencing of the DNA samples obtained from PCD patients exclusively lacking ODAs (as determined by TEM) revealed, among others, loss-of-function mutations in the following loci: *CCDC114* [MIM: 615038] [106,107,108], *CCDC151* [MIM: 615956] [109], *ARMC4* [MIM: 615408] [110,111], and *TTC25* [MIM: 617095] [112].

Immunofluorescence studies showed that, in human respiratory epithelial cells, proteins encoded by these genes localize throughout the ciliary axonemes. The loss-of-function mutations in all above loci have similar phenotypic outcomes. The HSVM analyses revealed that cilia are generally immotile or rarely display residual twitching or flickery movement. Immunofluorescence analyses using anti-DNAH5 and anti-DNAH9 antibodies confirmed the absence of ODAs [106,107,109,112]. The only exceptions are cilia in cells with mutated *ARMC4*, where ODAs are reduced but not completely absent (DNAH5 and DNAI2 are present in the proximal part of cilia). Consequently, some cilia exhibit reduced motility, but with significantly reduced frequency and amplitude [110].

TTC25 seems to be the most upstream and ARMC4 the most downstream player in the ODA docking cascade. While TTC25 is present in cilia lacking DNAH5, CCDC114, CCDC151, or ARMC4 [112], CCDC114, CCDC151, and ARMC4 are undetectable in cilia of respiratory epithelial cells obtained from individuals with a loss-of-function mutation in *TTC25* [112]. In turn, CCDC114 and ARMC4 are undetectable in the respiratory cilia of the epithelial cells from individuals carrying a *CCDC151* mutation [109], and ARMC4 is not present in *CCDC114* mutants, while CCDC114 is detected in cilia of *ARMC4* mutants [110]. The immunoprecipitation analyses of tagged proteins expressed in HEK293 cells revealed that CCDC114 co-immunoprecipitates with TTC25 [112] and CCDC151 [109]. Moreover, TTC25 and CCDC151 can interact with IFT subunits [113,114]. Based on these data, it was proposed that TTC25 is indispensable for either the transport of ODA docking complexes into cilia or for the attachment of the other ODA docking proteins to the axoneme in cilia assembled by human respiratory cells [112].

The genomes of unicellular models such as *Chlamydomonas* and *Tetrahymena* encode proteins with similarity to CCDC114 and CCDC151 (ODA10 in *Chlamydomonas*), while they lack orthologs of TTC25 [112] and ARMC4 [110]. In *Tetrahymena*, the CCDC114 and CCDC151 proteins have not yet been analyzed. In *Chlamydomonas*, the ODA docking complex is composed of three proteins: ODA1 (an ortholog of CCDC114) and *Chlamydomonas*-specific ODA3 and ODA14 [115,116,117,118]. *Chlamydomonas* ODA10 and another protein, ODA5, are cytoplasmic proteins and interact with the preassembled dynein arms before they are transported to flagella, and *Chlamydomonas* mutant *oda10* lacks ODAs [119]. Thus, in the case of ODA docking proteins, genetic and microscopic analyses using mice and zebrafish embryos are invaluable to better understand the distribution and role of these proteins in ciliated cells, and consequently, to reveal the cause of cilia immotility and PCD. The analyses of zebrafish embryos with knocked down or mutated genes encoding ARMC4 [110], CCDC115 [109,113], or TTC25 [114] and mice mutants [109,110,112] confirmed that these proteins are required for the presence of ODAs in cilia. Moreover, in pronephric cilia of the zebrafish embryo, the presence of Ccdc114 in cilia depends upon the presence of Ccdc151 [113], similar to the case of PCD patients with mutations in *CCDC151* [109]. Some authors also reported the assembly of fewer and shorter cilia in *ttc25* zebrafish [114] and *Xenopus* [120,121] mutants, although these data are contradicted by other groups [112].

PCD manifested exclusively by ODAs defects can also be caused by mutations in two other genes: *MNS1* (meiosis specific nuclear structural 1) [MIM: 610766] [122,123] and *CCDC103* [MIM: 614677] [124,125]. A loss-of-function mutation in *MNS1* reduces the number of the assembled ODAs (usually only 5–7 ODAs are visible in cilia cross-section) and leads to the laterality defect (situs inversus) and male infertility [122]. MNS1 co-immunoprecipitates with CCDC114, suggesting that it plays a role in ODA docking complex assembly or stability [122] or the stable binding of ODAs to the docking complex.

CCDC103 is a coiled-coil domain-containing protein which has been shown in *Chlamydomonas* to bind tightly to the microtubules of the peripheral microtubular doublets along their entire length with a periodicity of 12 nm [126]. Interestingly, immunofluorescence analyses of the respiratory epithelial cells obtained from patients harboring mutations in *CCDC103* revealed that DNAH5 and DNAI2 are lost from the distal part of cilia (indicating ODAs defects), but are still detectable in the proximal part. In agreement, the DNAH9 specifically present only in the distal part of cilia is undetectable in cilia assembled by respiratory cells carrying *CCDC103* mutation [124]. The immunofluorescence localization data are consistent with TEM observations showing either a lack or reduction of the ODAs in cilia cross-sections [124,125]. As in the case of mutations in other genes affecting ODA assembly, cilia in individuals with a loss-of-function mutation in *CCDC103* are immotile [124,125]. The biochemical studies in *Chlamydomonas* and zebrafish embryos showed that CCDC103 can form a stable dimer and is associated with the axoneme even when ODAs are missing [124].

#### 5.3.3. Factors Involved in Dynein Arms Preassembly

Strikingly, in a significant number of PCD cases that manifest by a lack of the outer and inner dynein arms (as determined by TEM analyses), the conducted genetic tests failed to reveal mutations in genes encoding subunits of these structures. The elucidation of this puzzle has led to the discovery of (1) the cytoplasmic proteins (called dynein axonemal assembly factors, DNAAFs) that act as co-chaperons during the preassembly of the dynein arms, and (2) proteins that play a role in the preassembly and/or transport of ODAs and IDAs to cilia. To date, mutations in the following genes were shown to cause PCD with ultrastructural changes described above: *DNAAF1/LRRC50* (leucine-rich repeat containing) [MIM: 613190] [127,128], *DNAAF2/KINTOUN* (*KTU*) [MIM: 612517] [129], *DNAAF3/C19orf51* [MIM: 614566] [130,131], *DNAAF4/DYX1C1* (dyslexia susceptibility 1 candidate 1) [MIM: 608706] [132,133], *DNAAF5/HEATR2* (HEAT-repeat-containing protein 2) [MIM: 614864] [134,135], *DNAAF6/PIH1D3* [MIM: 300933] [136,137], *CFAP300/C11orf70* [MIM: 618058] [138,139,140], *LRRC6* [MIM: 614930] [141,142,143], *SPAG1* [MIM: 603395] [144], *DNAAF7*/*ZMYND10* [MIM: 607070] [145,146,147], and *CFAP298/C21orf59/FBB18/KURLY* [MIM: 615494] [148]; for review, see [25]. 

Individuals with PCD caused by the mutation(s) in one of the above-listed genes have immotile cilia that either lack the outer and inner dynein arms or have severe defects in these structures, as was revealed by TEM and immunofluorescence analyses using dynein arm marker proteins. The requirement of these proteins for dynein arms assembly was confirmed by studies conducted in different model organisms, including *Chlamydomonas*, *Trypanosoma*, *Paramecium*, *Drosophila*, zebrafish, and mouse [127,129,134,135,138,146].

Dynein arms and ODA docking complexes are preassembled in the cell body and transported independently to cilia by IFT (intraflagellar transport) particles [149]. To date, the data concerning interactions between DNAAFs, DNAAFs and dynein arms subunits, as well as DNAAFs and heat shock protein chaperones, are fragmentary. It is proposed that DNAAFs assist heat-shock protein chaperones in the folding and pre-assembly of these complexes [150,151]. 

### 5.4. Nexin–Dynein Regulatory Complex (N-DRC)

The N-DRC (earlier named the nexin link) was first described as a bridge-like structure connecting two adjacent outer microtubular doublets [152,153]. To date, mutations in three genes encoding N-DRC subunits (DRC proteins, dynein regulatory complex proteins) have been shown to cause PCD. These are *DRC1/CCDC164* [MIM: 615288] [154,155], *DRC2/CCDC65* [MIM: 611088] [148,156], and *DRC4/GAS8* (growth arrest specific, earlier named *GAS11*) [MIM: 605178] [157,158,159]. In contrast to evident structural alterations in the cilia of PCD patients carrying mutations in either *CCDC39* or *CCDC40* genes or gene encoding proteins indispensable for the assembly of the functional ODAs, cilia in individuals with PCD caused by the mutation in genes encoding N-DRC subunits at first glance have normal or nearly normal structure [154,155,160]. However, a careful examination of cilia cross-sections reveals a lack of the nexin links, a slight reduction of the number of IDAs, and, in some cilia, misaligned peripheral doublets [148,154,157,158]. The identification of such minor alterations in cilia requires detailed TEM examination and an experienced diagnostician. Moreover, one has to keep in mind that, even in healthy individuals, cilia with misaligned peripheral doublets occur and that not all N-DRC are well visible on cilia cross-sections (mostly only one to four nexin links can be detected per ciliary cross-section) [161,162].

Although difficult to detect, the ultrastructural alterations in N-DRC cause apparent changes in cilia beating. The high-speed video microscopy of nasal ciliated respiratory cells obtained by nasal brush biopsy from patients with PCD caused by the mutation in *CCDC164* or *CCDC65* revealed that, in affected individuals, cilia beat with a higher frequency than in healthy individuals [89,154,156]. Moreover, cilia seemed to be stiff and hyperkinetic, and their beat amplitude was slightly reduced [148,154,155]. The increase of cilia beating frequency was not observed in cases of PCD caused by the mutations in *GAS8*. In these patients, only a slight reduction of the bending amplitude was reported [157,158]. 

The number of antibodies specifically recognizing DRC subunits is limited. Immunofluorescence studies showed that cilia of the respiratory cells obtained from patients with PCD caused by mutations in *CCDC164* lacked GAS8 [154,155] and DRC3/LRRC48 (see below) [154] and that DRC3 is not present in respiratory cells obtained from patients with a mutation in *GAS8* [157].

The genetic, biochemical, and microscopic analyses of the *Chlamydomonas* mutants shed light on the molecular basis of the cilia dysfunction in the affected individuals [163,164,165,166,167,168]. In green algae, the N-DRC is composed of 11 (DRC1–11) highly evolutionarily conserved proteins [164] and forms numerous connections with other main ciliary complexes within the 96 nm axonemal unit and with the adjacent peripheral doublet [163]. It was proposed that N-DRC (1) restricts the extent of the outer doublet sliding and thus plays a role in the converting sliding of the outer doublets into an axonemal bend, and (2) functions as a main hub that regulates and coordinates the activity of the complexes in the axonemal unit [163]. Consequently, the lack of the N-DRC affects the function of several other axonemal structures. 

As was shown in *Chlamydomonas*, DRC1/CCDC164, DRC2/CCDC65, and DRC4/GAS8 proteins form a core of the N-DRC [166,167,168]. The localization of DRC1 and DRC2 is interdependent, and a loss-of-function mutation in genes encoding either protein results in the loss of both DRC1 and DRC2, as well as other DRC subunits, DRC5, 6, and 11, and the reduction of the level of DRC3 and DRC7–10—summarized in Table 1 in [168]—and some inner dynein arms [163]. As a consequence, a substantial portion of the N-DRC is missing, as was confirmed by the cryo-ET analyses of the *Chlamydomonas* mutant flagella [163]. In contrast to the phenotype observed in patients with *CCDC164/DRC1* mutation, flagella in *Chlamydomonas* DRC1 mutants beat with a normal or slightly reduced frequency [100]. 

In *Chlamydomonas*, the *DRC4* loss-of-function mutation does not affect the ciliary localization of DRC1 and DRC2 [164,165,167,168], but DRC3/LRRC48, DRC5/TCTE1 (t-complex-associated-testis-expressed 1), DRC6/FBXL13 (F-box and leucine-rich repeat protein 13), and DRC7/CCDC135 are missing [164,165]. 

Less is known about the other DRC subunits. The N-DRC complex in the flagella of *drc3 Chlamydomonas* mutant lacks only DRC3, which builds a distal part of the complex [169]. The *drc3* mutants swim only slightly slower than wild-type cells (about 80% of the wild-type) and mutant flagella beat with a higher frequency (about 130%) but lower beat amplitude [169]. Significantly, mutation in DRC3 causes PCD in mouse model (Table 1) [170]. 

In *Chlamydomonas*, limited ultrastructural changes are also caused by the mutations in genes encoding DRC5. Flagella of *Chlamydomonas* with mutations in DRC5 (*sup-pf-4* mutant) lack only two N-DRC subunits: DRC5/TCTE1 and DRC6/FBXL13 [164]. Thus, it is possible that, besides *CCDC164/DRC1*, *CCDC65/DRC2,* and *GAS8/DRC4*, mutations in other genes encoding N-DRC subunits can cause PCD in humans.

### 5.5. Radial Spokes

The individuals with PCD caused by the mutations in genes encoding components of the radial spokes, similar to individuals with mutation in proteins building the central apparatus (see below), have all symptoms of the disease except for laterality defects (nodal cilia lack radial spokes and a central apparatus, as was mentioned above).

The vast majority of the pioneering work that aimed at the identification of the radial spoke protein composition and localization of the individual subunits within these structures was carried out in *Chlamydomonas* [171,172,173,174,175,176,177,178], for review [179], and tunicate *Ciona intestinalis* [180,181,182]. 

Morphologically, radial spokes resemble a capital letter “T” and can be divided into three parts: A stem that is attached to the peripheral microtubule doublet and extends toward the central apparatus; a head, the most distal part of the radial spoke that can interact with central apparatus projections; and a neck, which connects a stem and head. Out of 23 radial spoke proteins identified in *Chlamydomonas*, 12 seem to have orthologs in humans, and mutations in these genes can most likely cause motile cilia dysfunction. Indeed, mutations in five genes encoding radial spoke proteins have been reported to cause PCD in humans. These are genes encoding subunits of the radial spoke head: *RSPH1* [MIM: 609314] [183,184,185], *RSPH4A* [MIM: 612647] [186,187,188], and *RSPH9* [MIM: 612648] [186,189,190,191]; a subunit of the radial spoke stem—*RSPH3* [MIM: 615876] [192]; and, based on the analyses in model organisms, a subunit of the radial spoke neck—*RSP16*/*HSP40*/*DNAJB13* [MIM: 610263] [193]. 

The high-speed video microscopy of nasal epithelial cells from individuals carrying mutations in *RSPH4A* and *RSPH9* revealed that cilia beat with a slightly lower frequency and display abnormal circular movement. In the case of *RSPH1*, *RSPH3,* and *DNAJ13B/HSP40* mutations, cilia not only beat with a slightly lower frequency, but also with reduced amplitude. The analyses using classical TEM demonstrated that more than half of the cilia cross-sections seem to have normal (9 + 2) organization, while the remaining cilia lack the central apparatus (9 + 0) or have a shifted outer doublet to the cilium center (8 + 1). Additionally, in patients with an *RSPH3* mutation, the lack of the entire radial spokes is apparent. Interestingly, more detailed analyses of cilia of nasal epithelial cells obtained during biopsy from individuals carrying mutations in *RSPH1* revealed that only radial spokes RS1 and RS2 lack their head part, while the structure of the radial spoke RS3 seem to be intact [194]. Thus, most likely, the protein composition of RS3 and RS1/RS2 is at least partly different.

The structural alterations observed in the respiratory cilia from individuals carrying mutations in radial spokes proteins are in agreement with data obtained during the analyses of *Chlamydomonas* mutants. Wild-type *Chlamydomonas* cells have two spokes of a normal size, RS1 and RS2, while the third spoke is reduced to a short knob-like structure. *Chlamydomonas* cells with mutations in genes encoding the RSP3 protein, an ortholog of RSPH3, lack the entire RS1 and RS2 and have paralyzed flagella, while the flagella assembled by the RSP4 mutants (*Chlamydomonas* RSP4 is an ortholog of human RSPH4A) lack only the radial spokes’ head and move abnormally [195,196]. The depletion of the *HSP40/DNAJ13B* in *Chlamydomonas* causes minor structural defects in the neck of RS1 and RS2, but these two complexes are affected to a different extent [197]. Flagella of the mutated cells move in an uncoordinated way, generally without cells propelling [198]. It was proposed that HSP40, besides its function as a chaperone, stabilizes the distal part of the docked radial spokes [197].

### 5.6. Central Apparatus

Although it is commonly accepted that part of the signals regulating cilia beating originate at the central apparatus, surprisingly, mutations in only three genes encoding the components of the central apparatus, *HYDIN* [MIM:610812] [199], *SPEF2* [MIM:610172] [200], and *CFAP221* [201], were so far identified as a cause of PCD in humans. In PCD patients, a loss-of-function mutation does not affect (*HYDIN*) or only slightly reduces (*SPEF2, CFAP221*) the beating frequency of the respiratory cilia, but cilia movement is uncoordinated, with reduced bending and beating amplitude. Some immotile cilia are also observed [199]. The TEM analyses of these cilia revealed the generally normal (9 + 2) organization of the axonemes [199,200]. However, more detailed analyses using electron tomography revealed a lack of C2b projection in the central apparatus in cilia of the respiratory cells obtained from individuals carrying mutation in *HYDIN* [199]. Mutations in *Hydin*, *Spef2,* and *Cfap221* genes caused symptoms typical for PCD also in mice [202,203,204,205]. 

The detailed structural studies of the flagella of *Chlamydomonas* mutants corroborate the above data and reveal that HYDIN is a component of C2b projection, and its mutation results in the lack of C2b and the neighboring C2c projection [206]. CPC1, the *Chlamydomonas* ortholog of SPEF2, is a component of C1b projection, and mutation in CPC1 results in lack of C1b projection; importantly, CPC1 mutant cilia frequently lack also either part or entire C2b projection, suggesting that C1b stabilizes C2b projection [207]. PCDP1, a *Chlamydomonas* ortholog of CFAP221, is a component of C1d projection [208,209].

Using next-generation sequencing, mutations in two more genes encoding central apparatus proteins were found in individuals with PCD. The identified genes encode SPAG16, an ortholog of *Chlamydomonas* PF20, and SPAG17, orthologous to *Chlamydomonas* PF6. Based on the studies in *Chlamydomonas*, PF20 is a component of the bridge-like structure connecting two central microtubules [210], while PF6 is a component of a C1a projection [211]. In affected individuals, the mutations in *SPAG16* and *SPAG17* were accompanied by the mutation in *LRRC6* gene (see Section 5.3.3). Because TEM images were not provided, it is not clear if lack of the dynein arms was the only ultrastructural defect in these individuals [212]. Importantly, mice with mutated *Spag17* develop PCD symptoms, including hydrocephalus, the accumulation of mucus in the sinuses, and severe respiratory distress. Moreover, they could not suckle and died within 12 h of birth, likely from respiratory failure. The TEM analyses of the tracheal cilia in these mutant mice revealed the absence of either one of the central microtubules or a projection of a C1 microtubule [213]. Other potential PCD candidate genes are likely to be described soon [200].

PCD is also caused by the mutation in serine/threonine kinase *STK36*/*FU*/*FUSED* [MIM: 607652] [214]. The TEM analyses of the respiratory cells from PCD patients showed that the vast majority of respiratory cilia cross-sections have normal (9 + 2) organization, and the abnormalities in the central apparatus can only occasionally be found. However, cross-sections at the level of basal bodies revealed that basal feet (the basal foot is a structure associated with the basal body, required for the polarization of basal bodies [215]) are not properly aligned [214]. 

In normal respiratory cells, STK36 localizes along the entire cilia length. Interestingly, STK36 is not detected in cilia of the respiratory cells obtained from patients with mutations in genes encoding radial spoke subunits RSPH1, RSPH4A, and RSPH9, suggesting that the presence of the fully assembled radial spokes is required for STK36 ciliary localization [214]. Misoriented basal bodies were also observed in Fu−/− mice, but in contrast to cilia in PCD patients, assembled cilia frequently lacked the central apparatus [216]. Moreover, the biochemical data obtained during the analyses of murine ortholog of STK36 showed that Fused/Stk36 co-immunoprecipitates with Spag16/Pf20 and Pcdp1/Cfap221, the component of the bridge-like structure and subunit of the C1d central apparatus projection, respectively, and with kinesin Kif27 located at the cilium base [216,217]. The significance of the interactions between Stk36 and Kif27 is not clear; some hypotheses are discussed by Nozawa and co-authors [217]. Based on the above data, it is proposed that STK36 could connect two ciliary complexes, the central apparatus and radial spokes, and likely participates in the transduction of signals regulating cilia beating [214].

## 6. Other Proteins Causing PCD-Like Symptoms in Humans

The whole-exome or genome sequencing of samples obtained from the individuals with clinical features consistent with PCD led to the identification of mutations in additional loci, and thus additional likely PCD-related genes. Among them are *GAS2L2* (growth arrest-specific protein 2-like 2) [218], *OFD1* [219,220], *RPGR* [221,222], and possibly *TEKT1* [223] and *LRRC56* [224].

GAS2-like 2 (growth-arrest-specific 2), together with GAS2-like 1 and GAS2-like 3, belong to the GAS2 family. All members of the GAS2 family facilitate interactions between actin filaments and microtubules, but the molecular mechanism behind these interactions is most likely protein type-specific. GAS2L2 is proposed to mediate the co-alignment of actin filaments and microtubules through interactions with EB proteins [225]. In ciliated HBE (human bronchial epithelial) cells obtained from healthy individuals, GAS2L2 localizes near basal bodies and actin fibers [218]. A loss-of-function mutation in *GAS2L2* does not visibly affect the cilia ultrastructure, but causes the random orientation of the basal feet and randomized ciliary beating. Additionally, the ciliary beat frequency is increased [218]. At the moment, it is not clear how GAS2L2 contributes to the orientation of the basal bodies in multiciliated cells. 

*LRRC56* is another PCD candidate gene. Its mutations do not visibly affect the cilia ultrastructure as determined by TEM, but cilia are severely dyskinetic [224]. The molecular mechanism underlying cilia dysfunction is not clear. When co-expressed in HEK293 cells, LRRC56 co-immunoprecipitates with IFT88, suggesting its role in ciliary transport [224]. Bonnefoy and co-authors suggest that human LRRC56 is an ortholog of *Chlamydomonas* ODA8. However, human LRRC56, a 542 amino acid protein, and the much larger protein *Chlamydomonas* ODA8 (921 amino acids) and a *Trypanosoma* ortholog show similarity only within approximately the 100 amino acid region containing LRR domains. The depletion of the LRRC56-like protein in *Trypanosoma* affects the assembly of the ODAs, but only in the distal part of the axoneme [224]. Flagella assembled by *Chlamydomonas oda8* mutants lack ODAs; it is proposed that ODA8 participates in the maturation and transport of ODAs to flagella [226]. Thus, ODA8 is either not a true ortholog of LRRC56 or, if these proteins are orthologous, they play different roles in human cilia and in the flagella of the unicellular organisms. 

OFD1 (oral–facial–digital type I) protein localizes to the distal part of the centriole and basal bodies and is involved in the control of centriole length and the assembly of the distal appendages [227]. Until now, the mutations in *OFD1* were associated with ciliopathies caused by defects in the primary cilia and manifested, among others, by severe neurological and skeletal abnormalities (e.g., oral–facial–digital syndrome type 1 [228], Jourbert syndrome [229]). Interestingly, newly identified mutations in exons 20 and 21 cause symptoms characteristic for PCD, without the severe skeletal or neurological symptoms typical for other *OFD1* mutations [219]. Respiratory epithelial cells obtained from patients carrying newly identified mutations assemble fewer but surprisingly much longer cilia. The TEM analyses revealed that a significant number of basal bodies remain in the cell body, suggesting defects in their docking to the apical cell surface (which is in agreement with OFD1 being present in the distal appendages of the basal body). The HSVM analyses revealed the different degree of cilia motility defects in different patients; from immotile cilia, stiff cilia beating asynchronously with reduced amplitude to nearly normally beating cilia [219]. The altered beating of the respiratory cilia was also reported in individuals with the frameshift mutation in the exon 16 of the *OFD1* gene [230]. These observations are in agreement with data showing that OFD1 localizes to the basal bodies in human nasal epithelial cells [231].

Human OFD1 isoform 1 is a 1012 amino acid protein. A search of the genomes of *Chlamydomonas*, *Tetrahymena,* and *Paramecium* (our unpublished data and [232]) resulted in the identification of proteins with some similarity to human OFD1, but only in the N-terminal region. The depletion of OFD1 in the ciliate *Paramecium* impaired the docking of the basal bodies to the cell cortex [232]. In zebrafish embryos, the depletion of OFD1 caused changes in the phenotype consistent with the defects of both motile and primary cilia [233]. 

Mutations in *RPGR* (retinitis pigmentosa GTPase regulator) cause the degeneration of the retina (retinitis pigmentosa). However, similar to the case of *OFD1*, some mutations in *RPGR* also seem to affect motile cilia and cause symptoms typical for PCD besides vision problems [222]. The RPGR has two main isoforms: The so-called constitutive form of RPGR^EX 1–19^ (transcript consists of 19 exons, 815 amino acids) and the RPGR ORF15 isoform that contains exons 1–14 and alternatively spliced exon 15 and intron 15 (1152 amino acids). The RPGR ORF15 is present specifically in the connecting cilium of rods and cones. The RPGR^EX 1–19^ was also detected in the connecting cilium of the photoreceptors, but additionally in the primary cilia [234] and in the transition zone of motile cilia in airway epithelia [235]. The functional importance of RPGR is not fully understood. RPGR can interact with a number of ciliary proteins, including transition zone proteins (e.g., NPHP4, RPGRIP1L) [236,237,238], suggesting its role in the regulation of the ciliary trafficking. The morpholino-driven knock-down of *Rpgr* in zebrafish causes the assembly of shorter cilia [239,240]. On the other hand, it was suggested that defects in motile cilia function in PCD patients are caused by the misorientation of the assembled cilia [221]. 

Recently, a case of a patient was reported, who, besides having symptoms typical for Mainzer–Saldino syndrome (MZSDS) such as nephronophthisis, retinal dystrophy and skeletal abnormalities [241], also suffered from recurrent airway infections [223]. The HSVM analyses of the cells obtained from nasal brushing revealed that the number of cilia was significantly reduced, and assembled cilia were either immotile or beat with a reduced amplitude and with very low frequency [223]. The TEM studies showed that basal bodies in the analysed multiciliated cells were misoriented, while ciliary axonemes seemed to have a normal ultrastructure [223]. The exon-enriched NGS led to the identification of the mutations in two genes: (1) *WDR19*, encoding the intraflagellar transport component IFT144, known to be associated with ciliopathies caused by the improper function of the primary cilia [242]; and (2) *TEKT1*, which earlier was not associated with ciliopathies. Tektin-1 localizes to the centrosome, basal bodies, and along the motile cilia axoneme, but not in the primary cilia [223]. 

*WDR35/IFT121* encoding an intraflagellar transport protein is an example of another gene whose mutations were previously associated only with ciliopathies caused by the dysfunction of the primary cilia, such as cranioectodermal dysplasia. It is worth mentioning that immotile or dyskinetic respiratory cilia causing respiratory problems were observed in a patient with cranioectodermal dysplasia caused by a mutation in *WDR35* [243]. 

## 7. Other Proteins Causing PCD in Model Organisms: Novel Candidate Genes in Humans?

It is estimated that among patients with diagnosed PCD, in approximately 30% of cases, the mutated causative genes have not been identified. Interestingly, studies of the ciliary proteins using vertebrate models led to the identification of additional genes causing the phenotypic alteration typical for PCD in these models (Table 1). These genes could be good candidates for PCD-causative genes in humans. 

## 8. Summary

The following statement can be found in one of the PCD-dedicated reviews: “Genetic studies involving human patients and mouse models of primary ciliary dyskinesia over the last decade have uncovered a number of important ciliary proteins and have begun to elucidate the mechanisms underlying ciliary motility. When combined with genetic, biochemical, and cell biological studies in *Chlamydomonas reinhardtii*, these mammalian genetic analyses begin to reveal the mechanisms by which ciliary motility is regulated” [248]. This statement can be safely re-phrased: The combination of genetic, biochemical, and cell biological data obtained using model organisms allows us to elucidate not only the mechanisms that underlie cilia assembly and functions, but also to understand the basis of PCD in humans at the molecular level. 

The truth is that the research conducted in model organisms and data from affected individuals are like two opposite ends of the string; both lead to the string center, which is the full understanding of cilia biology and their impact on human health. Both the model organisms and human data are complementary to each other, and both have contributed tremendously to our current knowledge of the molecular mechanisms that regulate motile cilia assembly and function. Such hand-in-hand studies have already led to a better understanding of the basis of human disease and more advanced methods of PCD diagnosis, and hopefully, in the future, may lead to the development of appropriate therapies. 

## Figures and Tables

**Table 1:** Genes associated with PCD-like syndrome in vertebrate model organisms.

Mutated Gene	Model Organism	Localization in Cilia	Phenotype	Ref
*AK7*	Mouse	n/d	Reduced ciliary beat frequency, significant number of cilia lacking CA (9 + 0), or with displaced peripheral doublet without CA (8 + 1) or with CA; hydrocephalus, mucus accumulation in the paranasal passages, exacerbated respiratory responses upon allergen challenge, male infertility, situs inversus not detected	[244]
*CFAP54*	Mouse	C1d projection (based on studies in *Chlamydomonas*) [208,209]	Reduced ciliary beat frequency, lost C1d projection hydrocephalus, male infertility, and accumulation of mucus in the sinuses	[245]
*SPAG6*/*PF16*	Mouse	Central apparatus (based on studies in *Chlamydomonas*) [246]	Reduced ciliary beat frequency, asynchronous beating, reduction in cilia density, normal axoneme structure but random orientation of CA hydrocephalus, male infertility random orientation of basal feet of the basal bodies	[247]
*c15orf26*/*CFAP161*	Zebrafish	n/d	Missing outer dynein arms, pronephric cysts, axis curvature, laterality defects, hydrocephalus	[148]
*LRRC48*/*FAP134*/*DRC3*	Mouse	N-DRC (based on studies in *Chlamydomonas*) [164]	Hydrocephalus, laterality defects, male infertility, accumulation of mucus in the sinuses	[170]

AK7: Adenylate kinase 7; CA: Central apparatus; CFAP: Cilia- and flagella-associated protein; PCDP: Primary ciliary dyskinesia protein; PF: Paralyzed flagella; SPAG: Sperm-associated antigen; n/d: Not determined.

## Supplementary Materials

The following are available online at https://www.mdpi.com/2073-4409/8/12/1614/s1, Table S1: Genes associated with primary ciliary dyskinesia.

## Abbreviation

MIM, Mendelian Inheritance in Man; based on data from https://omim.org/.
