# Supplementary material for: Rare Human Diseases: Model Organisms in Deciphering the Molecular Basis of Primary Ciliary Dyskinesia
Source: Cells. 2019 Dec 11;8(12):1614. doi: 10.3390/cells8121614 (PMC6952885; doi:10.3390/cells8121614)
Supplement: Supplementary file 1 [file cells-08-01614-s001.pdf]

**Table S1**

Genes associated with primary ciliary dyskinesia

| Gene name | | | | | Comments |
|---|---|---|---|---|---|
| *H. sapiens* | *M. musculus* | *D. rerio* | *Aliases* | *C.reinhardtii (mutant strain)* protein | |
| *MCIDAS* | *Mcidas* | *mcidas* | | ---- | transcription co-regulator |
| *CCNO* | *Ccno* | *cyclin O* | | ---- | cytoplasmic protein; controls amplification of centrioles |
| *CCDC39* | *Ccdc39* | *ccdc39* | *CFAP59* | *FAP59* *(pf8)* FAP59 | likely defines 96-nm axonemal unit and docking site for N-DRC, IDAs and RSs |
| *CCDC40* | *Ccdc40* | *ccdc40* | *CFAP172* | *FAP172* *(pf7)* FAP172 | likely defines 96-nm axonemal unit and docking site for N-DRC, IDAs and RSs |
| *DNAH5* | *Dnah5* | *dnah5* | | *ODA2* *(pf28)* HC1γ | ODAs γ dynein heavy chain |
| *DNAH9* | *Dnah9* | *dnah9* | | *ODA4* *(oda4)* HC1β | ODAs β- dynein heavy chain |
| *DNAH11* | *Dnah11* | *LOC567790* | | *ODA4* *(oda4)* HC1β | ODAs β- dynein heavy chain |
| *DNAI1* | *Dnai1* | *dnai1.1* *dnai1.2* | | *ODA9, DIC1* *(oda9)* IC78, IC80 | ODAs intermediate chain |
| *DNAI2* | *Dnai2* | *dnai2a* *dnai2b* | | *ODA6, DIC2* *(oda6)* IC69, IC70 | ODAs intermediate chain |
| *DNAL1* | *Dnal1* | *dnal1* | | *DLC1, DLU1* LC1 | ODAs light chain |
| *NME8* | *Nme8* | *nme8* | *TXNDC3* | *DLC3, DLX1* LC3 *DLC5, DLX2* LC5 | ODAs light chain |
| *CCDC114* | *Ccdc114* | *ccdc114* | | *DCC2, ODA1* *(oda1)* ODA-DC2 | ODA docking complex |
| *CCDC151* | *Ccdc151* | *ccdc151* | | *ODA10* *(oda10)* ODA10 | ODA docking complex |
| *TTC25* | *Ttc25* | *ttc25* | | ---- | ODA docking complex |
| *ARMC4* | *Armc4* | *armc4* | | ---- | ODA docking complex |
| *MNS1* | *Mns1* | *mns1* | | *FAP127* FAP127 | Possibly ODA docking complex |
| *CCDC103* | *Ccdc103* | *ccdc103* | ---- | *PR46b, CCDC103* CCDC103 | Possibly ODA docking complex |
| *DNAAF1* | *Dnaaf1* | *dnaaf1* | *LRRC50* | *ODA7, DAU1* *(oda7)* ODA7 | Cytoplasmic |

| | | | | | |
|---|---|---|---|---|---|
| *DNAAF2* | *Dnaaf2* | *dnaaf2* | *KTU*<br>*Kintoun*<br>*C14orf104* | *DAP1, MOT45*<br>*(pf13, mot45)*<br>PF13 | Cytoplasmic |
| *DNAAF3* | *Dnaaf3* | *dnaaf3* | *C19orf51* | *DAB1, PF22*<br>*(pf22)*<br>PF22 | Cytoplasmic |
| *DNAAF4* | *Dnaaf4* | *dnaaf4* | *DYX1C1* | *PF23*<br>*(pf23)*<br>DYX1C1 | Cytoplasmic |
| *DNAAF5* | *Dnaaf5* | *dnaaf5* | *HEATR2* | HEATR2<br>CHLRE_<br>09g395500v5 | Cytoplasmic |
| *PIH1D3* | *Pih1d3* | *pih1d3* | *Twister* | *TWI1*<br>*(twi1)*<br>PIH1D3 | Cytoplasmic |
| *ZMYND10* | *Zmynd10* | *zmynd10* | | *ZMYND10*<br>CHLRE_<br>08g358751v5 | Cytoplasmic |
| *SPAG1* | *Spag1* | *spag1a*<br>*spag1b* | | ---- | Cytoplasmic |
| *CFAP298* | *Cfap298* | *cfap298* | *C21orf59*<br>*kurly* | *FBB18, DAB2*<br>CFAP298 | Cytoplasmic |
| *LRRC6* | *Lrrc6* | *lrrc6* | *Seahorse* | *MOT47*<br>*(mot47)*<br>MOT47 | Cytoplasmic |
| *CFAP300* | *Cfap300* | *cfap300* | *C11orf70* | *FBB5*<br>FAP300 | Cytoplasmic |
| *DRC1* | *Drc1* | *drc1* | *CCDC164* | *DRC1, PF3*<br>*(pf3)*<br>DRC1 | nexin link |
| *CCDC65* | *Ccdc65* | *ccdc65* | *DRC2*<br>*CFAP250* | *DRC2*<br>FAP250 | nexin link |
| *GAS8* | *Gas8* | *gas8* | *DRC4*<br>*GAS11* | *DRC4, PF2*<br>*(pf2)*<br>DRC4 | nexin link |
| *RSPH1* | *Rsph1* | *rsph1* | | *RSP1*<br>RSP1 | radial spoke head |
| *RSPH4A* | *Rsph4a* | *rsph4a* | | *RSP4*<br>*(pf1)*<br>RSP4A | radial spoke head |
| *RSPH9* | *Rsph9* | *rsph9* | | *RSP9*<br>*(pf17)*<br>RSP9 | radial spoke head |
| *RSPH3* | *Rsph3* | *rsph3* | | *RSP3*<br>*(pf14)*<br>RSP3 | radial spoke stem |
| *DNAJB13* | *Dnajb13* | *dnajb13* | | *RSP16, HSP40*<br>*(pf33)*<br>RSP16 | radial spoke neck |
| *HYDIN* | *Hydin* | *hydin* | | *HYDIN*<br>HYDIN | central apparatus |

| | | | | | |
|---|---|---|---|---|---|
| *STK36* | *Stk36* | *stk36* | | CHLREDRAFT_<br>104702 | central apparatus<br>Limited similarity to *Chlamydomonas* protein |
| *SPEF2* | *Spef2* | *spef2* | | *CPC1*<br>*(cpc1)*<br>CPC1 | central apparatus |
| *CFAP221* | *Cfap221* | ---- | | *FAP221*<br>FAP221, PCDP1 | central apparatus |
| *RPGR* | *Rpgr* | *rpgra,*<br>*rpgra* | | ---- | possibly transition zone region |
| *OFD1* | *Ofd1* | *ofd1* | | *OFD1*<br>OFD1 | basal foot of the basal body |
| *GAS2L2* | *Gas2l2* | *gas2l2* | | ---- | near basal bodies |
| *TEKT1* | *Tekt1* | *tekt1* | | ---- | centrosome, basal bodies, axoneme |
| *LRRC56* | *Lrrc56* | *lrrc56* | | *ODA8, DLU2*<br>*(oda8, mot37)*<br>ODA8 | Unknown<br>Limited similarity to *Chlamydomonas* protein |
